# Magnitude and Temporal Variability of Inter-stimulus EEG Modulate the Linear Relationship Between Laser-Evoked Potentials and Fast-Pain Perception

**DOI:** 10.3389/fnins.2018.00340

**Published:** 2018-05-31

**Authors:** Linling Li, Gan Huang, Qianqian Lin, Jia Liu, Shengli Zhang, Zhiguo Zhang

**Affiliations:** ^1^School of Biomedical Engineering, Health Science Center, Shenzhen University, Shenzhen, China; ^2^Guangdong Provincial Key Laboratory of Biomedical Measurements and Ultrasound Imaging, Shenzhen, China; ^3^Department of Communication Engineering, Shenzhen University, Shenzhen, China; ^4^Experimental Center of Fundamental Teaching, Sun Yat-Sen University, Zhuhai, China

**Keywords:** pain prediction, cross-individual prediction, inter-stimulus EEG, single-trial analysis, machine learning

## Abstract

The level of pain perception is correlated with the magnitude of pain-evoked brain responses, such as laser-evoked potentials (LEP), across trials. The positive LEP-pain relationship lays the foundation for pain prediction based on single-trial LEP, but cross-individual pain prediction does not have a good performance because the LEP-pain relationship exhibits substantial cross-individual difference. In this study, we aim to explain the cross-individual difference in the LEP-pain relationship using inter-stimulus EEG (isEEG) features. The isEEG features (root mean square as magnitude and mean square successive difference as temporal variability) were estimated from isEEG data (at full band and five frequency bands) recorded between painful stimuli. A linear model was fitted to investigate the relationship between pain ratings and LEP response for fast-pain trials on a trial-by-trial basis. Then the correlation between isEEG features and the parameters of LEP-pain model (slope and intercept) was evaluated. We found that the magnitude and temporal variability of isEEG could modulate the parameters of an individual's linear LEP-pain model for fast-pain trials. Based on this, we further developed a new individualized fast-pain prediction scheme, which only used training individuals with similar isEEG features as the test individual to train the fast-pain prediction model, and obtained improved accuracy in cross-individual fast-pain prediction. The findings could help elucidate the neural mechanism of cross-individual difference in pain experience and the proposed fast-pain prediction scheme could be potentially used as a practical and feasible pain prediction method in clinical practice.

## Introduction

Pain is a subjective perception and is primarily assessed by means of self-report. Because the capacity to effectively report pain is limited in vulnerable population groups (e.g., babies and people with cognitive or communicative impairments), assessment of pain levels based on physiological signals has attracted a growing interest (Wager et al., 2013). For example, several pain prediction models based on functional magnetic resonance imaging (fMRI) have been developed to assess pain in healthy persons and patients with chronic pain (Wager et al., 2013; López-Solà et al., 2017). As compared to fMRI, electroencephalography (EEG) could capture a wealth of pain-related brain activities in a cheap and easy-to-use manner, so EEG-based pain assessment is a promising technique in clinical settings. The most commonly used pain-related EEG activities in research are laser-evoked potentials (LEP), and the strong relationship between the LEP amplitudes and the subjective pain intensity on a trial-by-trial basis has been well characterized (Garcíalarrea et al., 1997; Iannetti et al., 2005; Hu et al., 2014). Based on the singe-trial LEP-pain relationship, our previous study has been able to predict subjective pain ratings from single-trial LEP features and achieved an accuracy greater than 80% (Huang et al., 2013a). The relationship between EEG activities and pain rating in other pain paradigms has also been identified and been used to develop EEG-based pain prediction models. For example, based on the spectral characteristics of EEG before drug treatment, one study classified responders and non-responders and presented the application of EEG-based pain models in the prediction of analgesic effect (Gram et al., 2015).

An EEG-based pain prediction model can be achieved at within-individual level (the model is trained on and applied to the same individual) or at cross-individual level (the model is trained on a group of individuals but applied to different individuals) (Huang et al., 2013b). Cross-individual pain prediction is more desired in clinical practice but it normally has a lower accuracy than within-individual prediction (Huang et al., 2013b), because the relationship between brain activity and pain ratings could vary largely among individuals. Actually, each individual has a unique prediction model that links his/her neural activities and pain perception. The substantial cross-individual variability in pain experience has been well studied, and it could be attributed to genetic constitution, socio-cultural variables, and cognitive states (Coghill et al., 2003; Tracey and Mantyh, 2007).

Some studies have also explored neural correlates of cross-individual variability in pain experience. The modulating effect of EEG on perceived pain intensity and pain-evoked neural responses (such as LEP) has been well documented in literature (Babiloni et al., 2008; Zhang and Ding, 2010; Anderson and Ding, 2011; Lange et al., 2012) and studied in our previous work (Bai et al., 2016; Tu et al., 2016). For example, an individual's pain-evoked LEP responses are significantly correlated with his/her spontaneous EEG in terms of magnitude (Bai et al., 2016), and the magnitudes of pre-stimulus EEG alpha and gamma oscillations modulate the forthcoming pain perception and LEP amplitude (Tu et al., 2016). Besides the magnitude of EEG, the temporal variability of EEG is also found to play a key role in effective functioning of various sensory systems, including visual (Treisman, 1964), auditory (Galin, 1964) and somatosensory (Zotterman, 1953). But, it remains unknown whether temporal variability of EEG is related to pain perception. As for fMRI studies, it has been shown that temporal variability of fMRI Blood Oxygenation Level Dependent (BOLD) signals is correlated with cross-individual variability in pain perception (Rogachov et al., 2016). Actually, investigating the functions and mechanisms of temporal signal variability, or moment-to-moment variability, of neural activities has gradually gained popularity. Temporal variability of neural signals can be observed at every level of the nervous system (Faisal et al., 2008), and it is not merely noise but functionally meaningful. An increasing number of work has confirmed the role of temporal brain signal variability as an individual differences measure across cohorts and across tasks (Garrett et al., 2013b). Based on above-mentioned studies, we can conclude that the mean and temporal variability of EEG and fMRI in the resting-state could be correlated with subjective pain ratings as well as pain-related neural responses.

In this study, we hypothesize that the parameters of a single-trial LEP-based pain prediction model are correlated with features of inter-stimulus EEG (isEEG). We are interested in isEEG correlates because isEEG is readily available during LEP recording and it is not necessary to do extra experiments to collect resting-state EEG or potential correlates of other modalities, such as genomic data and socio-culture variables. Also, previous studies have shown that the features extracted from inter-stimulus brain signals and spontaneous brain signals shared some similarities (Fair et al., 2007; Ganger et al., 2015). We aim to explore isEEG features (in terms of magnitude and temporal variability of isEEG rhythms) that are correlated with an individual's LEP-pain model parameters. More precisely, for each individual we train a simple linear prediction model to link the intensities of perceived pain and corresponding LEP magnitudes and then correlate the model parameters (slope and intercept) with isEEG features. Toward this goal, we collected isEEG/LEP data from 34 healthy subjects in a laser-evoked pain experiment. Then, we extracted magnitude and temporal variability of isEEG and correlated them with parameters of LEP-pain models across individuals.

Further, we aim to utilize the isEEG correlates of the cross-individual variability in the LEP-pain model to guide the design of individualized LEP-pain prediction models. To this end, we proposed a scheme to individualize the parameters of the LEP-pain prediction model based on isEEG correlates to improve the accuracy of cross-individual pain prediction. More precisely, an individual's pain prediction model will be trained only from those individuals with similar isEEG correlates, and our results show that such a scheme can achieve higher accuracy in pain prediction.

## Materials and methods

### Participants

Thirty-four right-handed healthy undergraduates (21.6 ± 1.7 years; 17M/17F) were enrolled and the inclusion criteria include: (1) non-smokers; (2) no history of chronic pain; (3) no acute pain symptom up to 4 weeks before the experiment; (4) no currently use of any medication. Before the experiment, all participants were familiarized with the details of procedure and gave written informed consent. The study was approved by the local ethics committee. Note that this dataset has been used in previously published articles (Hu et al., 2014; Bai et al., 2016).

### Experimental design

Nociceptive stimulation was produced by an infrared neodymium yttrium aluminum perovskite (Nd:YAP) laser with a wavelength of 1.34 μm (Electronical Engineering, Italy). With this wavelength, laser pulse could activate nociceptive terminals in superficial skin (Iannetti et al., 2006). The stimulation site was located at the medial side of the dorsum of left hand, between the first and third metacarpus. A helium-neon laser pointed to the region to be stimulated and the laser beam was transmitted via an optic fiber with a preset diameter of 38 mm^2^. The duration of laser stimulation was fixed at 4 ms and the target region was shifted by more than 1 cm in a random direction to avoid sensitization or nociceptor fatigue.

For each trial, the intensity of perceived pain was evaluated using a numerical rating scale (NRS) from 0 (no pain) to 10 (pain as bad as possible). In this experiment, participants were asked to report the intensity of pain perception with 4 as the pinprick pain (typical sensation for Aδ fiber-related fast-pain) threshold (Magerl et al., 1999; Basbaum and Bushnell, 2009). Only fast-pain trials will be included in following data analysis since the N2-P2 amplitude increased significantly only when the subjective pain intensity was larger than 4 (Bai et al., 2016). Prior to EEG experiment, the tolerable highest energy of the laser stimulation was determined for each participant by increasing the energy in steps of 0.25 J, until an NRS rating of 8 was reported. For each participant, 12–15 different levels of laser energies (from 1 to the tolerable highest energy in the range of 3.75–4.5 J, in step of 0.25 J) were adopted and 10 laser pulses at each energy level were delivered, resulting in a total of 120–150 pulses. The number of laser energy levels was different between subjects, because of the fixed lower limit (1 J) and the variable upper limit (3.75–4.5 J). The order of energy levels was pseudorandomized and the inter-stimulus interval varied randomly between 10 and 15 s (rectangular distribution).

### EEG data acquisition and preprocessing

EEG experiment was carried out in a silent and temperature-controlled room. Participants were seated in a comfortable chair and wore protective goggles. The EEG data collection was performed with a 64-channel EEG cap (Brain Products GmbH, Munich, Germany). The sampling rate was 1,000 Hz and the passband was 0.01–100 Hz. The electrode impedances were kept lower than 10 kΩ. Electrooculographic (EOG) signals were simultaneously recorded using surface electrodes to monitor ocular movements and eye blinks.

EEG data were analyzed using EEGLAB (Delorme and Makeig, 2004) and in-house MATLAB scripts (MathWorks). Continuous EEG data were band-pass filtered from 1 to 30 Hz using FIR filters. EEG data were further corrected using independent component analysis (ICA) algorithm (Makeig et al., 1997; Delorme and Makeig, 2004; Onton et al., 2006). ICA components that were considered as purely or predominantly driven by artifacts (such as ocular artifacts, myogenic artifacts) were discarded based on visual inspection of power spectrum, time course and topography. The remaining components were re-referenced to nose.

### Feature extraction

The N2-P2 complex is the largest deflection in LEP, which is a negative-positive vertex potential with maximal scalp distribution over the central region (Iannetti et al., 2008). LEP was quantified by the N2-P2 amplitude (peak-to-peak), which was calculated as the absolute difference between the N2 and P2 peak amplitudes measured from the most negative and positive deflections between 150 and 500 ms after stimulus onset.

**Figure 1:**
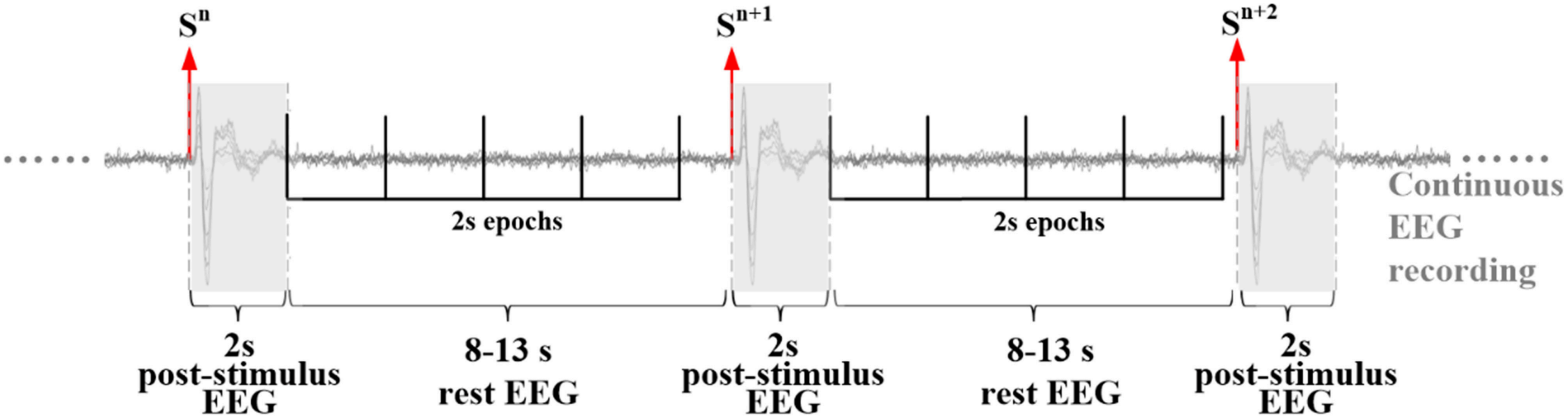
Illustration of extraction of isEEG epochs in the pain experiments. These 2s isEEG epochs were extracted from inter-stimulus EEG activity between laser stimuli.

The isEEG features (magnitude and temporal variability) were estimated from spontaneous EEG data recorded between adjacent laser stimuli. As shown in Figure 1, in the whole isEEG/LEP recording, we extracted a number of isEEG epochs from 2 s after each stimulus to the onset time of the next stimulus. Because the inter-stimulus interval varied randomly between 10 and 15 s, these isEEG epochs have a length ranging from 8 to 13 s. To avoid the influence of different data lengths on the estimation of isEEG features, these isEEG epochs were further segmented into isEEG epochs of 2 s so that each isEEG epoch could have 4–6 epochs. Next, we used random sampling with 100 repeated times to approximate the distribution of isEEG features. At each random sampling, we randomly selected one 2 s-epoch from each isEEG trial, and calculated its isEEG feature (magnitude or temporal variability), and then averaged the isEEG feature across epochs. After 100 times of random resampling, a distribution of isEEG features was obtained and its 50% percentile was used in subsequent data analysis procedures.

The magnitude of each isEEG epoch is quantified by root mean square (RMS):

(1) $$\begin{array}{r} \text{RMS}=\sqrt{\frac{1}{K}\sum_{k=1}^{K}S_{k}^{2}} \end{array}$$

where *s*_*k*_ is the *k*-th sample point of one isEEG epoch, and *K* = 2000 is the total number of time points of one isEEG epoch.

Temporal variability can be calculated in many forms, such as variance (He, 2011), standard deviation (SD) (Garrett et al., 2013a), mean square successive difference (MSSD) (Samanez-Larkin et al., 2010; Li et al., 2017). Here in this study, the temporal variability of isEEG was quantified by the normalized mean squared successive difference (nMSSD) (Neumann et al., 1941). Mean squared successive difference (MSSD) is a popularly used metric to characterize the temporal variability of physiological signals such as heart rate and fMRI BOLD signals (Berntson et al., 2005; Samanez-Larkin et al., 2010). Compared with other measures of temporal variability (such as SD), MSSD is not affected by low frequency drift and thus is more robust and reliable (Neumann et al., 1941; Li et al., 2017). Furthermore, because of the inherent relationship between signal strength and signal variability (i.e., the temporal variability calculated as MSSD or SD is positively correlated with the magnitude), MSSD should be normalized by RMS to disassociate the influence of signal strength (i.e., the magnitude) from signal variability. Hence, the normalized MSSD (nMSSD) was calculated as MSSD divided by the square of RMS:

(2) $$\begin{array}{r} \text{nMSSD}=\left[\frac{1}{K-1}\sum_{k=1}^{K-1}{\left(S_{k+1}-S_{k}\right)}^{2}\right]/RMS^{2} \end{array}$$

where *s*_*k*_ and *s*_*k*+1_ are the *k-*th and (*k* + 1)-th sample points one isEEG epoch, and *K* = 2000 is the total number of time points of one isEEG epoch.

The magnitude and temporal variability of isEEG (as quantified by RMS and nMSSD) were calculated for full-band isEEG waveforms as well as for band-limited isEEG waveforms at five frequency bands: delta (1–3 Hz), theta (4–7 Hz), alpha-1 (8–10 Hz), alpha-2 (11–13 Hz) and beta (14–30 Hz). The isEEG epochs were band-pass filtered with minimum-phase causal FIR filters to obtain waveforms at these five frequency bands.

### Relationship between isEEG features and LEP-pain model parameters

A linear regression model was fitted to investigate the single-trial relationship between pain ratings and LEP response for fast-pain trials. Note that, here we only consider fast-pain trials (NRS>4) because our previous study (Bai et al., 2016) has shown that significant correlation has only been observed between pain ratings and LEP responses of fast-pain trials. Actually, the magnitude of low-pain LEPs has no difference with that of isEEG.

For the *i*-th trial, the relationship between perceived pain rating and corresponding LEP magnitude could be described with a simple linear regression model as:

(3) $$\begin{array}{r} y_{i}=ax_{i}+b \end{array}$$

where *y*_*i*_ is the pain rating, *x*_*i*_ is the LEP magnitude, *a* and *b* are respectively the slope and intercept of the linear regression model. Therefore, for each participant, its individual LEP-pain model is characterized by two parameters: the slope *a* and the intercept *b*.

For a better interpretation of the model parameters, the linear model of (3) can be re-written as another equivalent linear model:

(4) $$\begin{array}{r} x_{i}=\left(\frac{1}{a}\right)y_{i}-\frac{b}{a}=cy_{i}+d \end{array}$$

where *c* = 1/*a* and *d* = –*b/a* are respectively the slope and intercept of the linear model (4). It can be seen clearly that, the slopes of two linear models (3) and (4) are reciprocals, and *d* in (4) is the “x-intercept” (where the linear line crosses the x-axis or the value of *x* when *y* = 0) in (3).

Subsequently, the correlation between isEEG features (RMS and nMSSD of full-band isEEG waveforms and isEEG at five frequency bands) and the parameters of LEP-pain model (4) was evaluated in order to identify isEEG correlates of the LEP-pain model parameters. Bonferroni correction is used to address the multiple comparison problem.

### Individualized LEP-pain prediction

Based on the identified isEEG correlates of the linear LEP-pain prediction model, we further develop a scheme for individualized fast-pain prediction. The basic idea is: an individual's pain rating will be predicted only using models from those individuals with similar distributions of model-related isEEG correlates. These isEEG correlates include magnitude and temporal variability of full-band isEEG and band-limited isEEG at each frequency band, and they were identified by the method in section of feature extraction. The scheme is detailed as follows.

1. A linear LEP-pain prediction model is trained within each individual, resulting in *N* prediction models.
2. Taking *m*-th individual as the test individual, his/her pain ratings can be predicted using each of the prediction models of other *N* – 1 training individuals. That is, the *i*-th LEP trial of the *m*-th individual has *N* – 1 predicted NRS values, denoted as $R_{m,i}^{\left(n\right)}$, *n* = 1, 2, …, *N* and *n* ≠ *m*.
3. The difference (measured as the Euclid distance) of isEEG correlates, *diff*_*n*_, is calculated between the test individual and the *n-*th training individual. A weight *w*_*n*_ is then calculated from *diff*_*n*_ and assigned to the predicted NRS values of the *m*-th individual from the *n-*th training individual. The weight is calculated as follows: (i) if *diff*_*n*_ is larger than the mean value across all *N* – 1 individuals, *w*_*n*_ is set to 0; (ii) otherwise, the weight is *w*_*n*_ = max(*diff*)–*diff*_*n*_, where max(*diff*) is the maximum value of all *N* – 1 *diff*_*n*_ values.
4. Finally, the pain rating of the *i*-th LEP trial of the *m*-th individual is calculated as the weighted average of $R_{m,i}^{\left(n\right)}$:
  (5) $$\begin{array}{r} R_{m,i}=\frac{1}{\sum_{w}}\sum_{\begin{array}{c} n=1\\ n\neq m \end{array}}^{N}w_{n}R_{m,i}^{\left(n\right)},with\sum_{w}=\sum_{\begin{array}{c} n=1\\ n\neq m \end{array}}^{N}w_{n} \end{array}$$

It can be seen from the proposed scheme that only individuals with similar values of isEEG correlates (i.e., individuals with similar LEP-pain relationship) are used to predict the pain ratings of the test individual, and the weight is inversely proportional to the distance of isEEG correlates between the test individual and the training individual.

Leave-one-individual-out cross validation was performed for the proposed individualized pain prediction scheme as well as for the conventional cross-individual pain prediction. To evaluate the prediction performance, Mean Absolute Error (MAE) was calculated as follows,

(6) $$\begin{array}{r} \text{MAE}=\frac{1}{T}\sum_{i=1}^{T}|R_{i}-{\hat{R}}_{l}| \end{array}$$

where *T* is the number of trials, *R*_*i*_ and ${\hat{R}}_{l}$ are the true and predicted pain rating for the *i*-th trial. The MAE values obtained from the proposed individualized pain prediction scheme and from the conventional cross-individual pain prediction model were compared using two-sample paired *t*-test.

## Results

### Relationship between LEP responses and pain ratings

**Figure 2:**
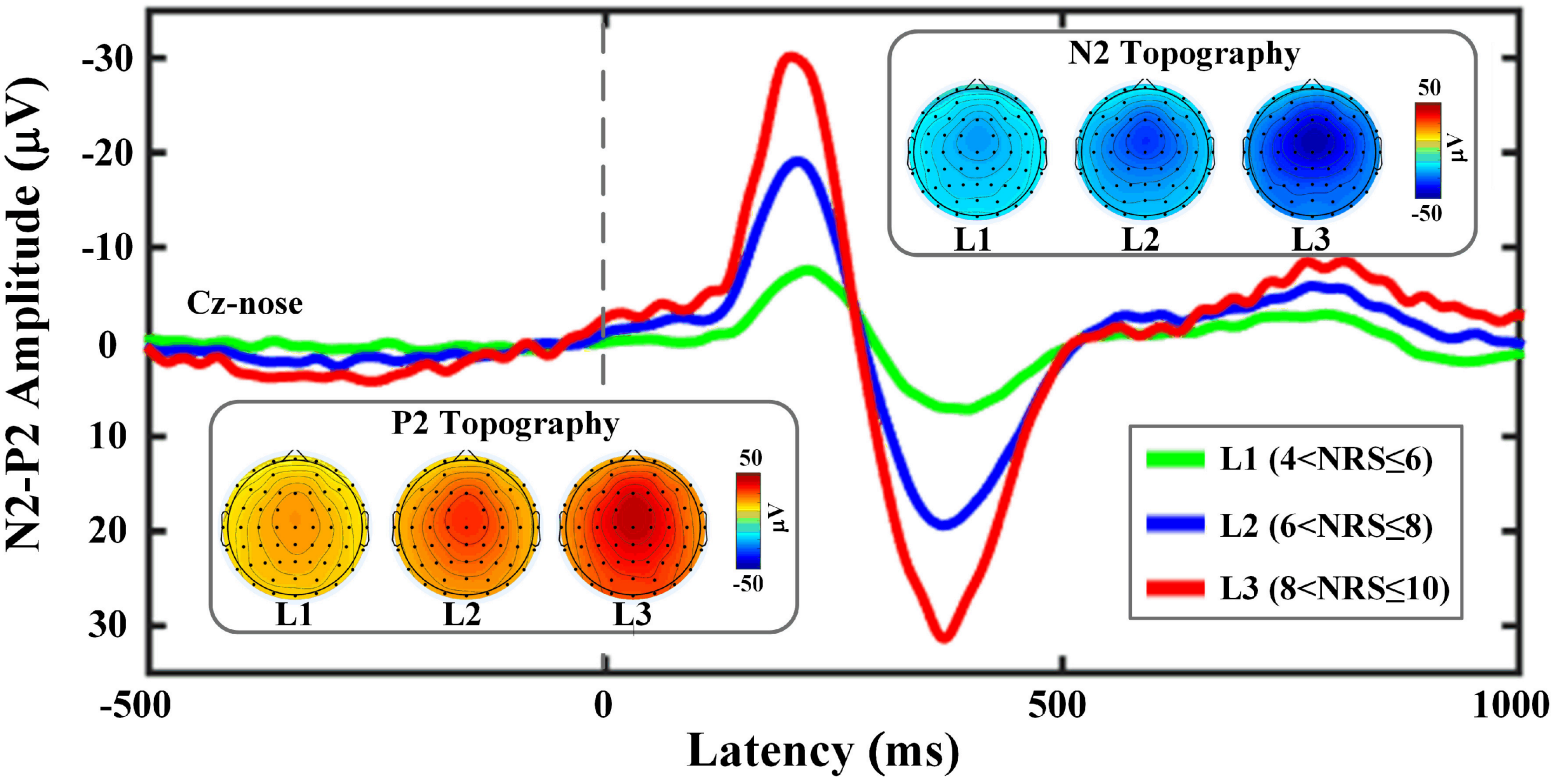
Group averages and scalp topographies of LEP responses at different NRS levels. LEP waveforms were recorded from the vertex (Cz-nose). The group mean value for N2 peak is −7.58 ± 8.88 μV, −19.06 ± 12.72 μV and −30.13 ± 18.84 μV for three levels respectively. The group mean value for P2 peak is 7.14 ± 8.20 μV, 19.38 ± 11.98 μV and 31.34 ± 12.70 μV for three levels respectively. The scalp topographies of N2 and P2 waves are displayed at their peak latencies.

**Figure 3:**
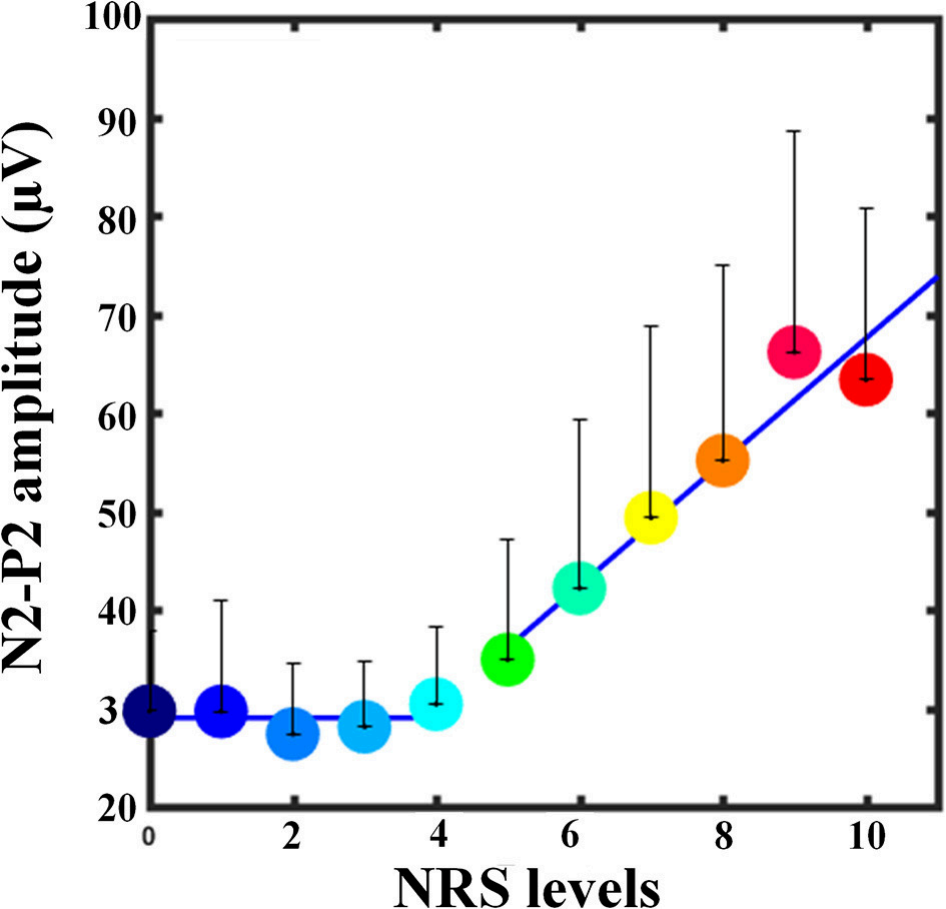
Relationship between pain intensity and N2-P2 amplitude at Cz. Colored dots represent the N2-P2 amplitudes of fast-pain trials averaged across participants, and error bars denote the SD values across participants. The blue line represents the fitted linear model for low-pain trials and fast-pain trials separately. Note that, when NRS = 10, the N2-P2 amplitudes are slightly lower than those of NRS = 9. The possible reason is that only 18 participants had fast-pain trials of NRS = 10, which might make the estimation have large variance and not reliable.

Two participants were discarded from the further analysis because of he/she only had fast-pain trials with NRS < 7. Figure 2 shows the group-averaged waveforms of the LEP responses elicited at Cz with three different NRS levels (4<NRS≤6, 6<NRS≤8, 8<NRS≤10), and the scalp topographies at the peak latencies of the N2 and P2 waves. When only fast-pain trials were included, linear correlation was observed between pain perception and corresponding brain responses (as shown in Figure 3).

### Relationship between isEEG features and LEP-pain model parameters

**Figure 4:**
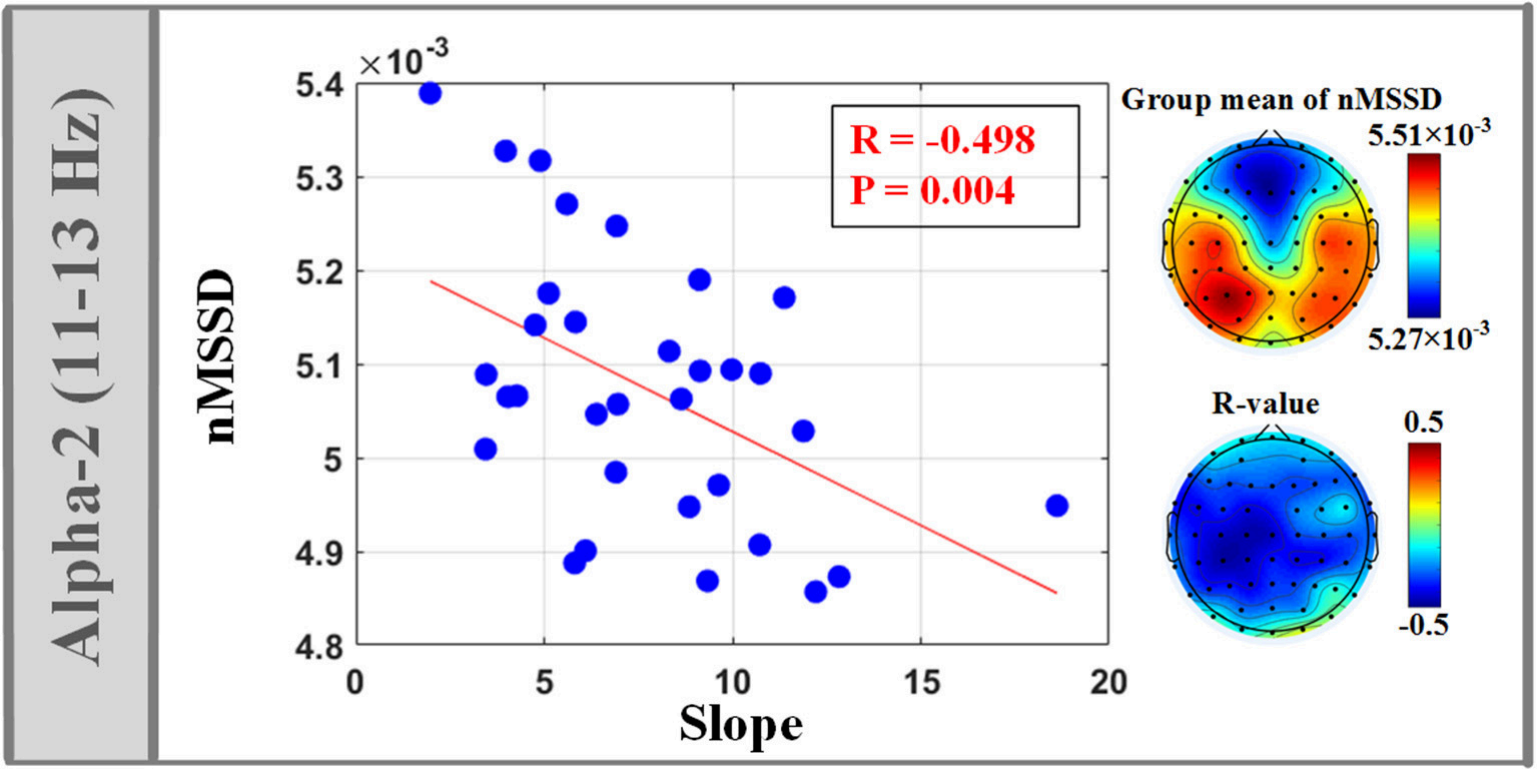
Significant correlation between temporal variability (nMSSD) of isEEG in alpha-2 band (11–13 Hz) and the fitting slope of the linear LEP-pain model (4).

**Figure 5:**
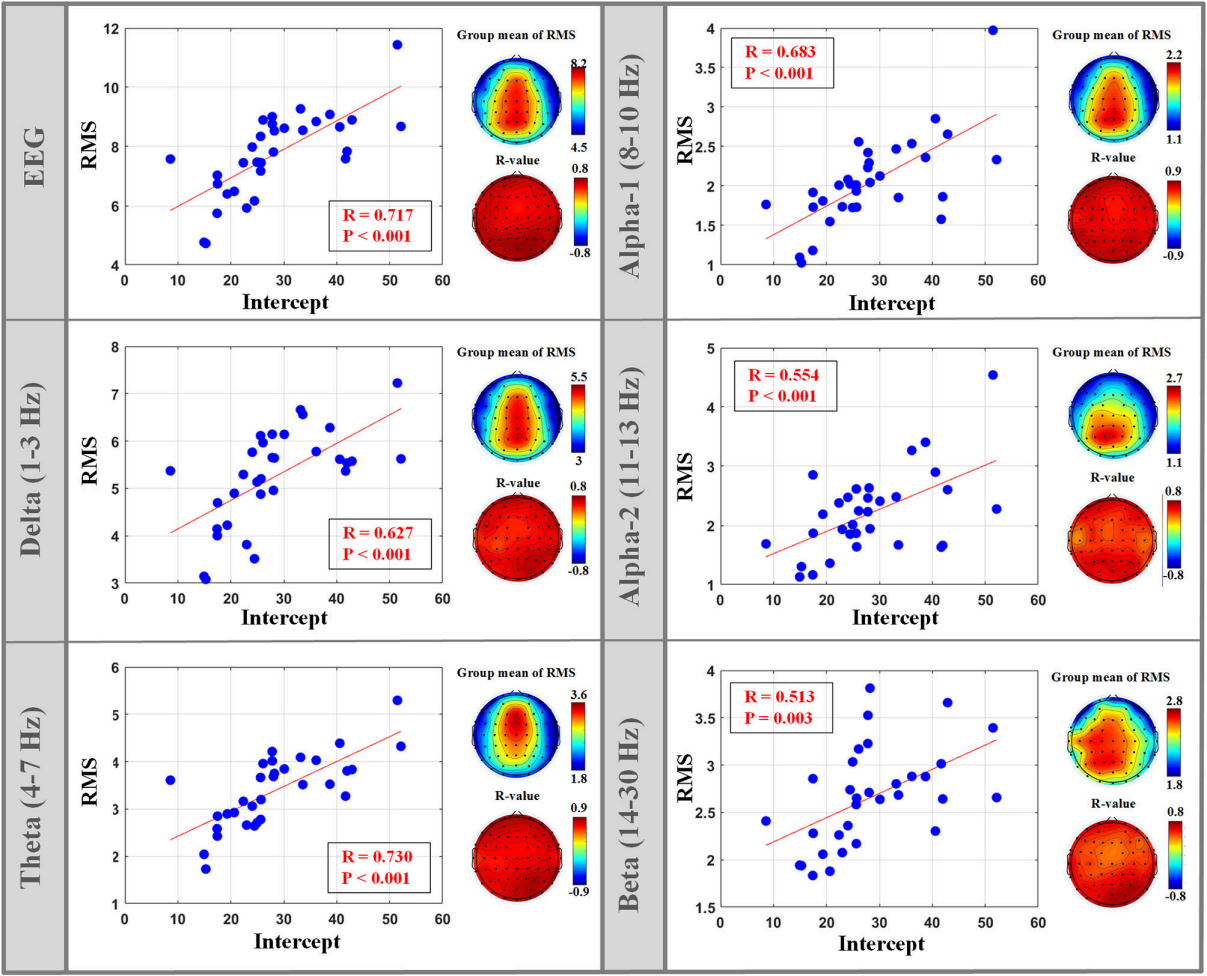
Significant correlation between isEEG magnitude (RMS) and the fitting intercept of the linear LEP-pain model (4).

The isEEG of alpha-2 band (11–13 Hz) across almost the whole brain showed significant negative correlation between nMSSD and the model slope of (4), as shown in Figure 4. Also, we observed that the model intercept of (4) showed significant positive correlation with RMS of isEEG across almost the whole brain in all frequency bands, as shown in Figure 5. Non-significant results are only reported in the Supplementary Material, as shown in Figure S1 and Figure S2.

### Individualized LEP-pain prediction model

The individualized LEP-based fast-pain prediction scheme can achieve a prediction error (MAE) of 1.19 ± 0.35 (mean ± SD), which is significantly smaller than the MAE, 1.36 ± 0.51, of the conventional cross-individual LEP-pain prediction (*p* = 0.002; two-sample paired *t*-test).

## Discussion

In this study, by analyzing isEEG data recorded during pain experiments from healthy participants, we obtained two main findings. First, both the magnitude (measured by RMS) and temporal variability (measured by nMSSD) of isEEG are related to cross-individual difference in the parameters of LEP-pain model for fast-pain prediction. Second, by selecting and weighting training individuals according to isEEG correlates of LEP-pain model, cross-individual fast-pain prediction accuracy could be significantly improved.

### Linear LEP-pain prediction model

The strong correlation between perceived pain intensity and the N2-P2 amplitude of LEP response at the single-trial level has been validated by numerous studies (Iannetti et al., 2008; Huang et al., 2013b; Hu et al., 2014). However, previous LEP studies usually adopted painful stimuli which can produce a clear pinprick pain sensation (Iannetti et al., 2008; Huang et al., 2013b; de Tommaso et al., 2017). Different from that, this study collected LEP data during stimulation with a wide range of energy intensities, including lower energy intensities which could not induce Aδ-related N2-P2 response. Based on this dataset, it has been shown that the linear relationship between pain ratings and LEP amplitude only exists for fast-pain trials (with NRS > 4) (Bai et al., 2016). Therefore, if the N2-P2 amplitude at Cz is used as the LEP feature to predict pain ratings of fast-pain trials, then the LEP-pain prediction model is defined by two parameters (slope and intercept). Different individuals have different LEP-pain relationship, so there is substantial cross-individual variability in the slope and intercept of the linear pain prediction models. A complex and nonlinear pain prediction model (other than the linear model) could be used for pain prediction, but it contains more model parameters whose meanings are difficult to be interpreted. So, we used a simple linear model to describe the relationship between single-trial LEP and pain rating.

### Positive correlation between isEEG magnitude and model intercept

Significant positive correlation was observed between RMS of isEEG and the intercept of the LEP-pain model for fast-pain prediction (4) at almost all frequency bands. Because the intercept is an inherent part of the LEP responses in (4) and the magnitude of LEP responses is positively correlated with isEEG magnitude for each individual (Bai et al., 2016), the intercept also has a positive relationship with isEEG magnitude. Actually, the positive relationship between the magnitude of isEEG and LEP has been observed in our earlier research from the same data set (Bai et al., 2016), which is due to anatomical factors (such as skull thickness and the orientation of the gray matter) and scale-electrode impedances.

### Negative correlation between alpha-2 isEEG temporal variability and model slope

Significant negative correlation between isEEG temporal variability (nMSSD) and the model slope was only observed in alpha-2 band (11–13 Hz). Alpha band activities are the dominant oscillations in the human brain, with a mean frequency of approximately 10 Hz. Growing evidence has shown that two independent alpha rhythmical components exist (alpha-1 and alpha-2 sub-bands) and they have different cortical sources and different physiological meanings and functional roles (Bazanova and Vernon, 2014). For example, their activities are different for dissimilar cognitive demands (Michels et al., 2008). Therefore, in pain research, alpha activities mediating the cognitive modulations of pain experience may display different activities in two alpha sub-bands. In this study, the significant correlation we observed in alpha-2 was maximal over ipsilateral and central electrodes. During the inter-stimulus period, alpha activities over central regions may mainly reflect the cognitive modulations such as anticipation and attention, since previous studies have revealed that the expectation period before the noxious stimulus is characterized by increased activations within pain-related cortical regions, including the bilateral anterior cingulate cortex, and medial prefrontal cortex (Keltner et al., 2006). Besides, as revealed in our previous study, pre-stimulus alpha activities, located bilaterally over central regions, significantly modulated the perceived pain intensity of subsequent stimulus and may partly reflect the neural activity of the sensory-motor network (Tu et al., 2016). Thus, alpha oscillation with greater dynamic range may represent better capacity for subject to engage antinociceptive and sensory-motor system and to manage their pain.

### Individualized cross-individual pain prediction using isEEG correlates

Compared with within-individual pain prediction, cross-individual prediction is more useful for clinical uses because it does not need subjective pain ratings for new individuals. However, the performance of cross-individual pain prediction is still not satisfactory because of the inherent cross-individual variability in pain perception. Cross-individual variability in pain perception could be observed in subjective pain ratings, pain-evoked brain responses or the relationship between them. In order to solve this problem for higher accuracy in cross-individual pain prediction, our previous study employed EEG-based normalization to incorporate individual traits that are related to cross-individual variability into the pain prediction model (Bai et al., 2016). The proposed normalization method was based on the stronger cross-individual correlation between the magnitude of isEEG and LEP (Bai et al., 2016). Here in this study, we observed that isEEG magnitude and temporal variability are related to cross-individual variability that in the LEP-pain model, and used isEEG correlates to tailor the parameters of the LEP-pain model to improve the accuracy of fast-pain prediction. The idea is to only use individuals with similar isEEG correlates to construct a LEP-pain prediction model for a new individual. The proposed method has achieved significantly lower estimation error in cross-individual pain prediction and could be potentially used as a practical and feasible pain prediction method in clinical practice.

## Limitations and future work

Proper assessment of pain is imperative for the development of an effective pain management plan, however, self-report pain is not available in some vulnerable populations (e.g., non-communicative patients with disorders of consciousness) (Schnakers et al., 2010). Therefore, the objective assessment of perceived pain from brain activity would be of enormous clinical implications (Huang et al., 2013b; Hu and Iannetti, 2016). In order to develop a more accurate and practical EEG-based pain prediction method, it is an important issue to reduce the error brought by cross-individual difference in pain (Fillingim, 2016). Characterizing the relationship between isEEG activities and LEP-pain prediction model parameters enables to capture isEEG correlates of cross-individual difference in the LEP-pain relationship, which is an important step to achieve individualized pain prediction. Here are some limitations of the current work and also outlines directions for future research. In this study, we observed isEEG features which were related to cross-individual differences in pain perception. However, much more factors, including genetic, environmental, psychological, and cognitive factors, may contribute to cross-individual differences in pain perception (Ochsner et al., 2006; Coghill, 2010). Future study with larger sample size and integrated dataset might find more kinds of features with significant correlation, then can make much more improvement in cross-individual pain prediction. Besides, we used isEEG data extracted from the inter-stimulus periods from nociceptive-stimulation experiment, which were inevitably affected by the periods with stimulation. Thus, future study could record resting-state EEG signal to validate the results we have achieved. Last but not least, advanced machine learning methods, such as transfer learning, could be applied to reduce cross-individual variability.

## Ethics statements

The protocol was approved by the ethics committee of Southwest University. All subjects gave written informed consent in accordance with the Declaration of Helsinki.

## Author contributions

LL, GH, and ZZ contributed to the construction of the study hypothesis. QL and JL collected the data. LL performed the data analysis and drafted the manuscript. Critical revisions were contributed by SZ and ZZ. All authors approved the manuscript for submission.

### Conflict of interest statement

The authors declare that the research was conducted in the absence of any commercial or financial relationships that could be construed as a potential conflict of interest. The reviewer RG and handling Editor declared their shared affiliation.
