# Supplementary figures and images for: Magnitude and Temporal Variability of Inter-stimulus EEG Modulate the Linear Relationship Between Laser-Evoked Potentials and Fast-Pain Perception
Source: Front Neurosci. 2018 May 31;12:340. doi: 10.3389/fnins.2018.00340 (PMC5991169; doi:10.3389/fnins.2018.00340)

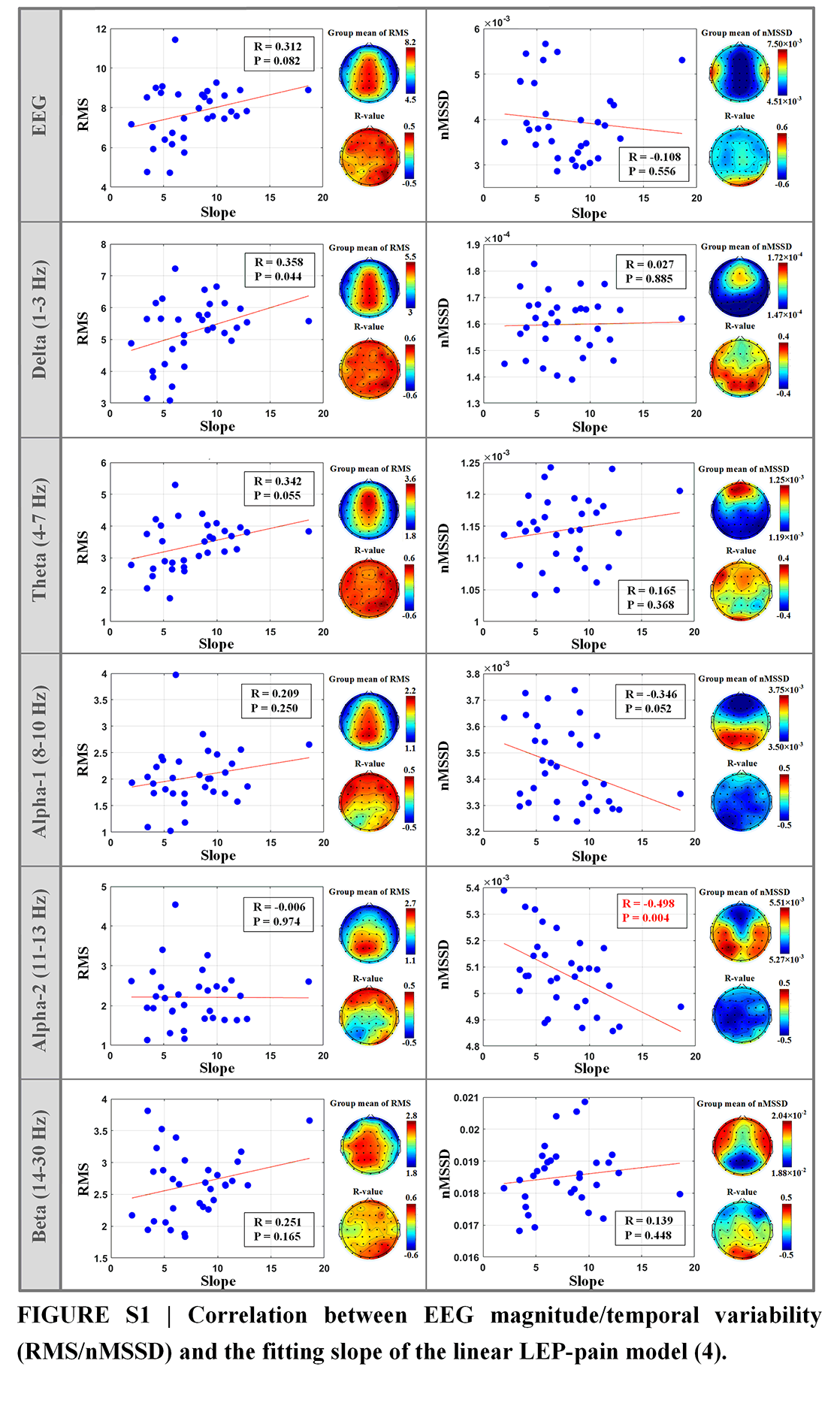

Supplement: Supplementary file 1 [file Image_1.TIF]

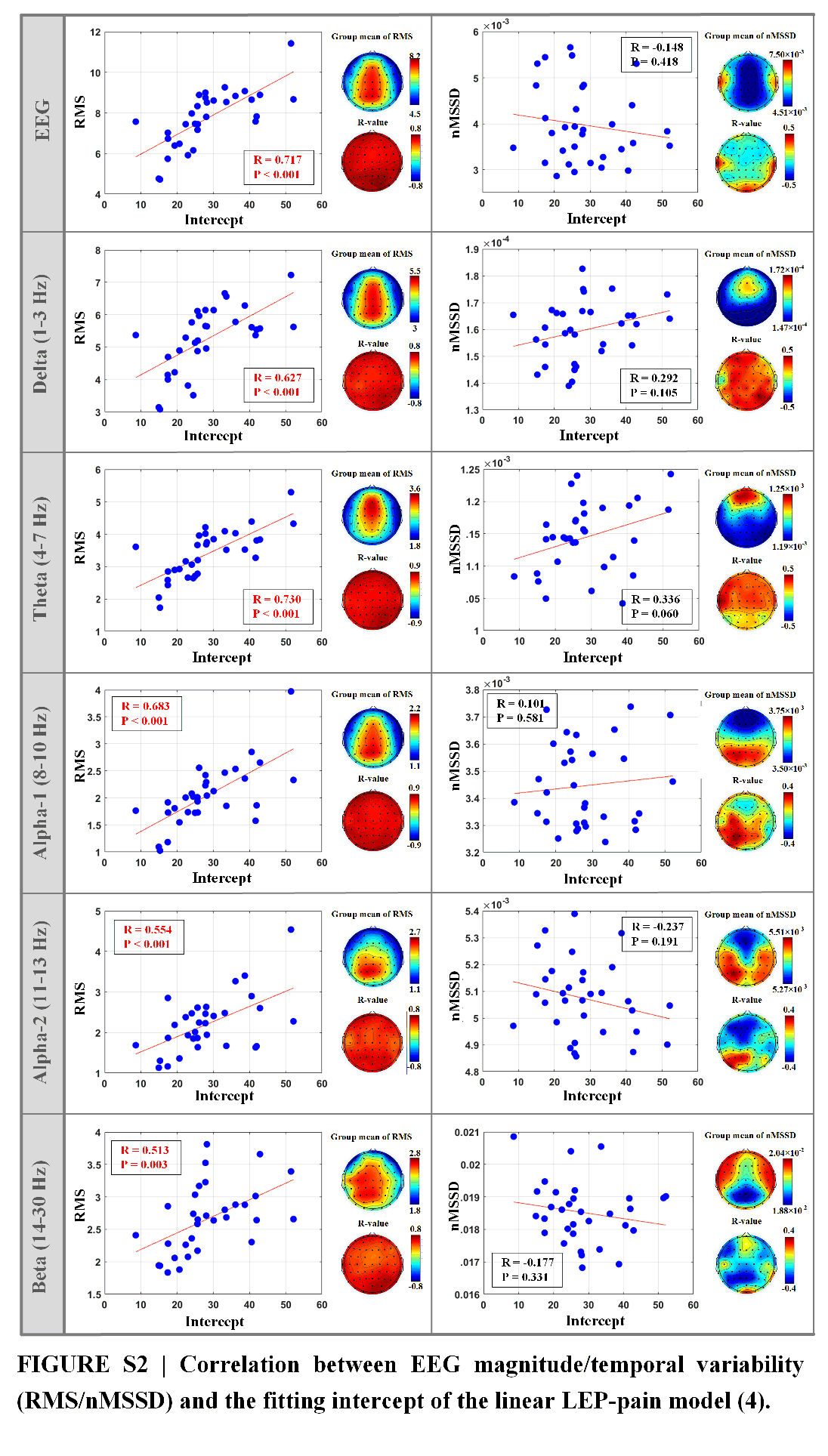

Supplement: Supplementary file 2 [file Image_2.TIF]
